# Towards Semantically-Rich Spatial Network Representation Learning *via* Automated Feature Topic Pairing

**DOI:** 10.3389/fdata.2021.762899

**Published:** 2021-10-20

**Authors:** Dongjie Wang, Kunpeng Liu, David Mohaisen, Pengyang Wang, Chang-Tien Lu, Yanjie Fu

**Affiliations:** ^1^ Computer Science Department, University of Central Florida, Orlando, FL, United States; ^2^ Computer Science Department, University of Macau, Macau, China; ^3^ Computer Science Department, Virginia Tech Falls Church, Falls Church, VA, United States

**Keywords:** feature-topic pairing, semantic space, spatial space, spatial representation learning, spatial graph

## Abstract

Automated characterization of spatial data is a kind of critical geographical intelligence. As an emerging technique for characterization, spatial Representation Learning (SRL) uses deep neural networks (DNNs) to learn non-linear embedded features of spatial data for characterization. However, SRL extracts features by internal layers of DNNs, and thus suffers from lacking semantic labels. Texts of spatial entities, on the other hand, provide semantic understanding of latent feature labels, but is insensible to deep SRL models. How can we teach a SRL model to discover appropriate topic labels in texts and pair learned features with the labels? This paper formulates a new problem: feature-topic pairing, and proposes a novel Particle Swarm Optimization (PSO) based deep learning framework. Specifically, we formulate the feature-topic pairing problem into an automated alignment task between 1) a latent embedding feature space and 2) a textual semantic topic space. We decompose the alignment of the two spaces into: 1) point-wise alignment, denoting the correlation between a topic distribution and an embedding vector; 2) pair-wise alignment, denoting the consistency between a feature-feature similarity matrix and a topic-topic similarity matrix. We design a PSO based solver to simultaneously select an optimal set of topics and learn corresponding features based on the selected topics. We develop a closed loop algorithm to iterate between 1) minimizing losses of representation reconstruction and feature-topic alignment and 2) searching the best topics. Finally, we present extensive experiments to demonstrate the enhanced performance of our method.

## 1 Introduction

Critical infrastructures (e.g., transportation networks, power networks, social networks, water supply networks) often consist of spatially distributed entities that interact with each other, and have generated massive spatial-networked behavior data. Analyzing such data can identify trends, forecast future behavior, and detect anomalies. To enable effective analysis, it is critical to desire a new capability of automated characterization that effectively extract feature vectors from spatio-networked data.

As one of the emerging techniques, representation learning can be adapted to learn non-linear embedded features of spatial network data, which we call spatial representation learning (SRL). There has been a rich body in SRL, including node embedding, autoencoder, random walk, adversarial learning, generative learning based methods with spatial data (Wang and Li, 2017; Wang et al., 2018a; Wang et al., 2018b; Chandra et al., 2019; Jean et al., 2019; Wang et al., 2019a; Wang et al., 2019b; Zhang Y. et al., 2019; Shan et al., 2020; Wang et al., 2020c; Wang et al., 2020d; Wang et al., 2021). Although these works achieved remarkable success, the model interpretability is still a big limitation that hinders these SRL methods from applying in more secure, fair, and rigorous scenarios.

Lacking model interpretability is possible to cause damaging or controversial consequences in incomplete scenarios that are not well-studied (Doshi-Velez and Kim, 2017). For instance, in the autonomous driving scenario, the end-to-end autopilot system brings high safety risks for drivers^1^. In 2015, Google’s photo app classifies images of black people as gorillas, which exposes the limitation of algorithms^2^. More seriously, widely used crime prediction software prefers to provide higher risk scores of future crimes for black defendants^3^. Model interpretability is one of the most important approaches to overcome these limitations. Thus how to enhance the model interpretability attracts much attention of researchers (Elshawi et al., 2019; Hong et al., 2020; Stiglic et al., 2020; Poursabzi-Sangdeh et al., 2021). But, many existing works reflect that there is a trade-off between model performance and model interpretability (Mori and Uchihira, 2019; Saisubramanian et al., 2020). Can we not only improve the model interpretability but also keep the model performance becomes the research point of this paper.

**FIGURE 1:**
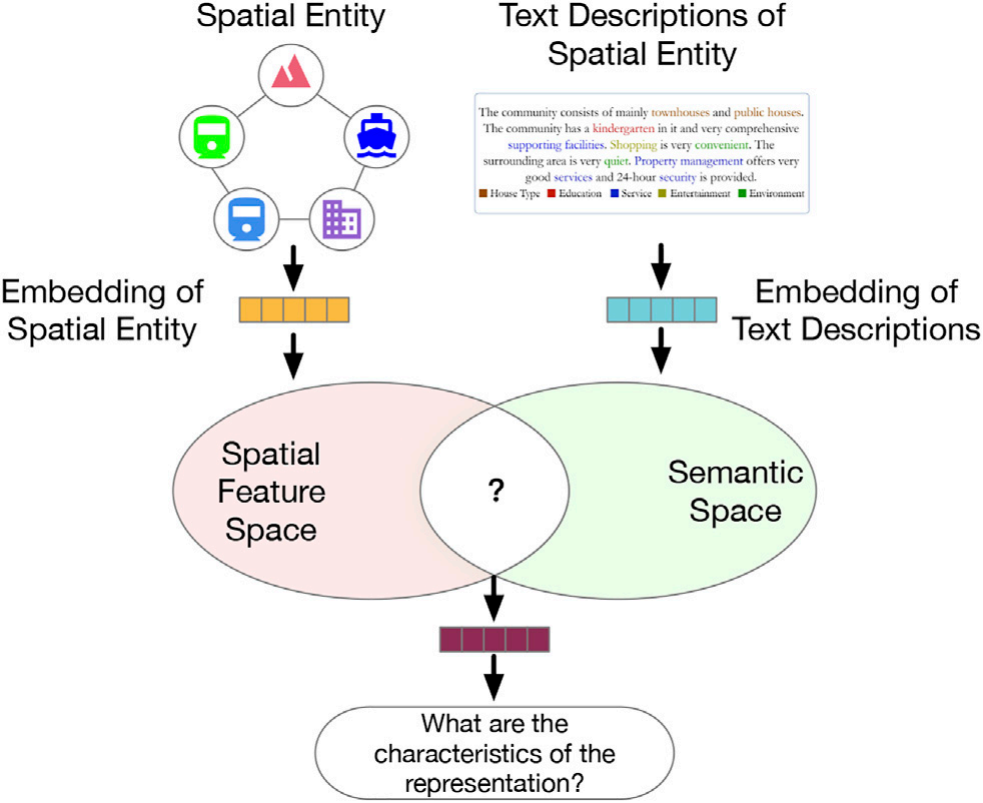
The motivation of the feature-topic pairing problem: bridging the gap between feature embedding space and topic semantic space in representation learning.

To relieve the limitations of prior literature and expand the application scenarios of SRL approaches, a novel SRL model should understand not just which features are effective, but also what these effective features stand for. This issue relates to two tasks: 1) deep representation learning; 2) label generation and matching for latent embedded features. In response, we formulate the problem as a task of feature-topic pairing (Figure 1), which is to align a latent embedding feature space, consisting of multiple latent features, and a textual semantic topic space, consisting of multiple topic labels during SRL. The basic idea is to teach a machine to extract topic labels from texts, and then pair the labels with learned features. To that end, we propose to develop a novel deep learning framework to unify feature learning, topic selection, feature-topic matching.

**FIGURE 2:**
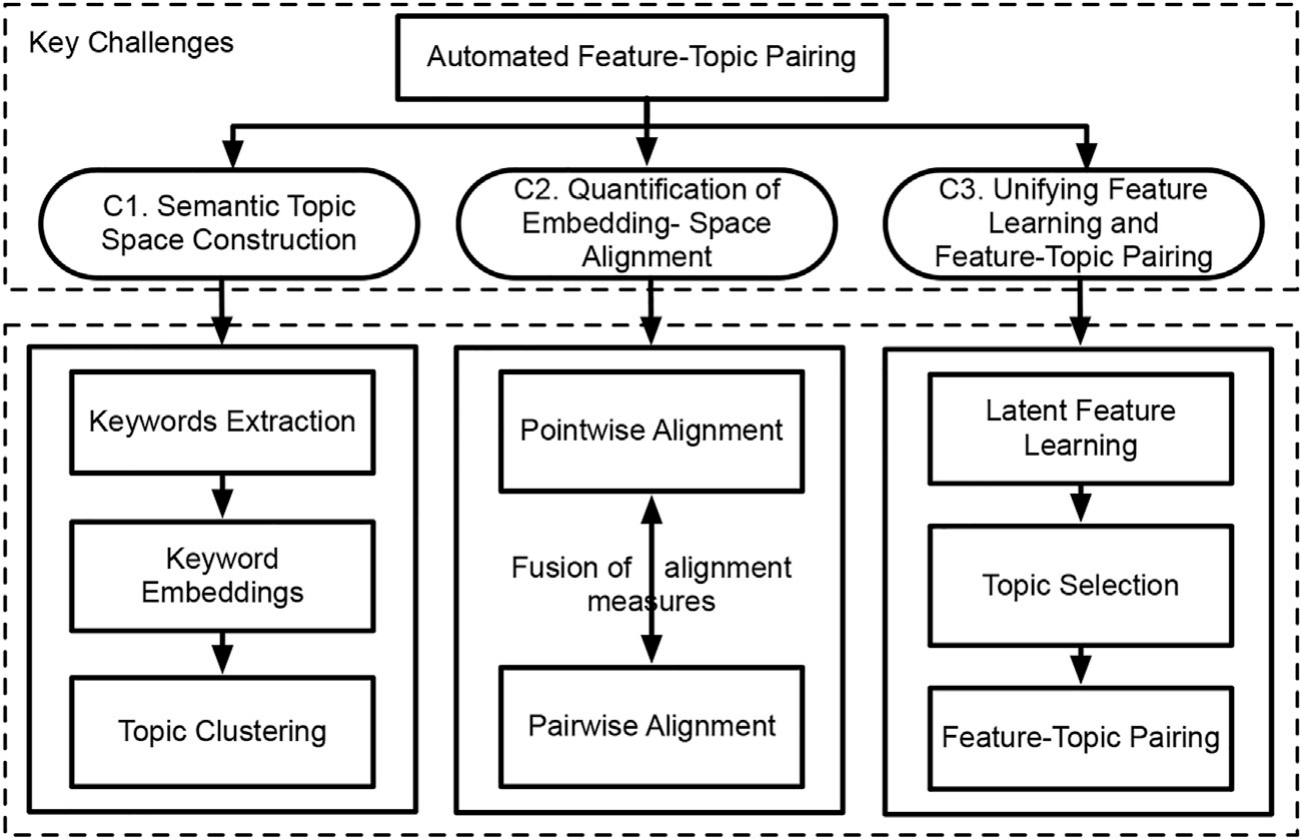
Key challenges and main tasks of the feature-topic pairing problem.

There are three unique challenges (Figure 2) in addressing this problem. 1) Label Generation Challenge. The semantically-rich texts of spatial entities describe their types, functions, and attribute-related information. For instance, on a real estate website, the texts of a residential community describe crime rates and events, great school ratings, nearby transportation facilities, grocery stores, companies, and universities. These texts, if properly analyzed, will help to identify which underlying features truly attract residents to pay more to live. However, these spatial texts are all unstructured, how can we construct a textual semantic topic space for spatial entities to support feature-topic pairing? 2) Measurement Challenge. Be sure to note that we aim to teach a machine to automatically perform the automated pairing between embedded features and topic labels in a self-optimizing fashion. As a result, a measurement is needed to quantify the alignment or matching score between the topic label space and the embedding feature space, in order to guide the machine about how to search. However, there is no standard measurement for quantifying the topic-embedding space alignment. Thus, what does form of measurement should be adopted? And how can we integrate the suitable measurement into the whole self-optimizing framework? 3) Optimization Challenge. Since the model needs to decide an optimized topic label subset, the feature-topic pairing problem evolves multiple machine learning tasks, including feature learning, topic label selection, and feature-topic matching. If the three tasks are separately completed step by step, there is no guarantee that they are globally optimized. So, how can we develop a deep optimization framework to jointly and simultaneously unify the three tasks?

To solve the three challenges, we develop a new PSO-based framework (named AutoFTP) that enclose the optimizations of feature learning, topic selection, and feature-topic pairing in a loop. Specifically, our contributions are:

1) Formulating the feature-topic pairing problem. Motivated by lacking feature labels in SRL, we formulate and develop a new problem: feature topic pairing. In the proposed model, we propose a new strategy: we first let an optimizer to automatically select K topics; the optimizer then guides the representation learner to learn K latent features that optimally align with the K topics.
2) Generating candidate topic labels. We propose a three step mining method to generate candidate topic labels. Specifically, we first extract keywords from the texts of all spatial entities. Then, we learn keyword embedding feature vectors with a pre-trained word model (He, 2014). Finally, we cluster all keyword embeddings by maximizing inter-topic distances and minimizing intra-topic distances to generate topics as candidate feature labels.
3) Quantifying feature-topic alignment measurement. We identify two types of feature-topic alignments: 1) point-wise alignment, and 2) pair-wise alignment. First, the point-wise alignment is to describe the correlation between an embedding feature vector and a categorical topic distribution. In particular, we maximize the correlation so that the distance between the distribution of the embedding vector space and the distribution of the topic semantic vector space can be minimized. The underlying motivation of point-wise alignment is originated from the fact that: if a topic density is high in describing a spatial entity, the topic’s corresponding feature value is expected to be large to co-vary with the topic density. In this way, we align the distribution covariance of the two spaces. Second, the pair-wise alignment is to describe the consistency between a feature-feature similarity matrix and a topic-topic similarity matrix. In particular, we use the feature-feature similarity graph to describe the topology of the latent embedding feature space, and use the topic-topic similarity graph to describe the topology of the textual semantic topic space. If the two spaces are aligned, the two graphs (represented by matrices) are similar as well.
4) Optimization in the loop. We develop a Particle Swarm Optimization (PSO)-based algorithm. In this algorithm, we first simultaneously optimize the representation learning loss, point-wise alignment loss, pair-wise alignment loss, and downstream task loss as the feedback for PSO. Guided by the feedback, the PSO based algorithm selects a better K-sized topic subset for feature-topic pairing. In particular, based on the loss function value, PSO iteratively generates topic masks (i.e., 0–1 indicators to select or deselect) to search the optimal topics for space pairing until the learning objective converges. In this way, the PSO jointly achieves topic selection, feature-topic pairing, and latent feature learning.

Finally, we evaluate our method using Beijing’s urban geography and mobility data. For comparison we implemented a broad range of other algorithms. Results showed that our method consistently outperformed the competing methods. We perform ablation study, interpretability, robustness check, stability, sensitivity to justify our technical insights.

## 2 Preliminaries and Problem Statement

In this section, we introduce key definitions of AutoFTP and the problem statement.

### 2.1 Particle Swarm Optimization

**FIGURE 3:**
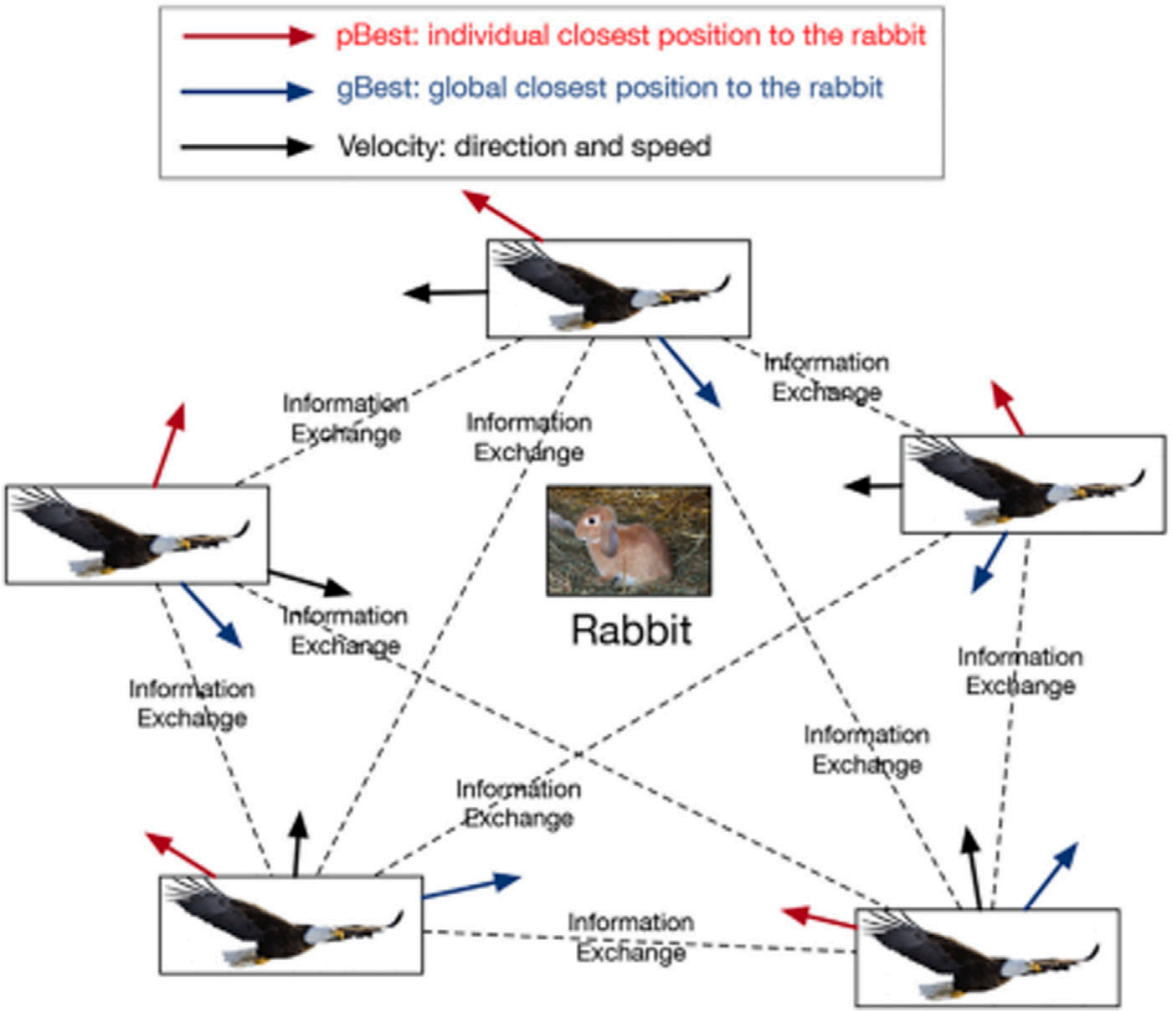
The origin of PSO: a flock of eagles is preying on a rabbit. To capturing the rabbit quickly, each eagle records where is the closest position to the rabbit during its history exploration (pBest). Meanwhile, all eagles share the closest position to the rabbit where the flock knew (gBest). All eagles explore any position based on their velocity, pBest, and gBest until the rabbit is captured.

PSO is a heuristic optimization algorithm that finds an optimal solution in a dynamic environment, by imitating the social activity of a flock of birds. Figure 3 shows the origin of PSO. A flock of eagles wants to capture a rabbit. To achieve the goal, all eagles exchange information related to the position of the rabbit. Each eagle updates its position based on its current status, velocity, the position where it knew is closest to the rabbit, and the position where the flock knew is closest to the rabbit, until the rabbit is captured.

Similarly, solving the feature-topic pairing problem can be analogized as a task of searching the optimal matching solutions in a dynamic environment. Specifically, we can view the eagles as a set of binary topic selector, which are to select the optimized subset of topics from a candidate topic set for feature-topic pairing. The choices of these binary topic selectors are iteratively updated in order to converge into the ultimate most matched topic-feature pairs. During the iterative process, all the binary topic selectors jointly share the changes of objective function losses (i.e., the losses of representation construction, feature-topic alignment, and downstream predictive task), so the topic selector knows how to update the topic selection next round.

### 2.2 Definitions

Definition 1: Spatial Entity. A spatial entity is a geographical concept that consists of a range (e.g. a circle area with a radius of 1 mile) and a location (i.e. the latitude and longitude of a center). The spatial entity also includes various Points-of-Interest (POIs) of different categories (e.g., buildings of education, shopping, medical, banking, etc.).

**FIGURE 4:**
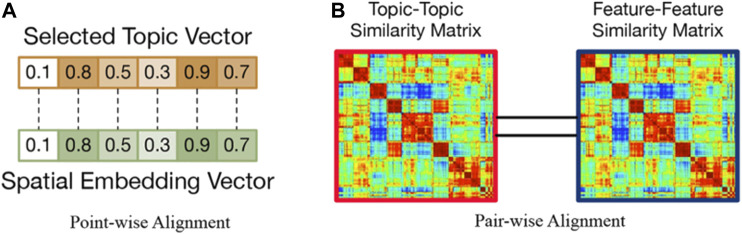
Two kinds of space alignment.

Definition 2: Point-wise Alignment. To tackle feature-topic pairing, we assume there are 1) an embedding vector that describes the features of a spatial entity and 2) a corresponding topic distribution associated to a spatial entity, which are extracted by optimization. To achieve feature-topic alignment, we propose a point-wise alignment to describe the correlation between features and topics. Figure 4A shows an example of point-wise alignment, we expect to maximize the correlation between the selected topic vector and the spatial embedding vector. The larger the correlation between the two vectors is, the larger the similarity between the two vectors larger is.

Definition 3: Pair-wise Alignment. We propose another perspective (pair-wise) to model the feature-topic alignment. For each entity-entity pair, we compute their feature-feature similarity and topic-topic similarity, and obtain: 1) a topic-topic similarity matrix **S**; 2) a feature-feature similarity matrix **S**′. We measure the consistency between the two matrices as the pairwise alignment.

Figure 4B shows an example of pair-wise alignment, we aim to let the topic-topic similarity matrix S as close as the feature-feature similarity matrix S′ possible.

### 2.3 The Feature-topic Pairing Problem

The feature-topic pairing problem aims to pair the latent features extracted by representation learning, with the explicit topics of texts of a spatial entity. Formally, given a set of *N* spatial entities, the *n*-th entity is described by multiple graphs (e.g., a POI-POI distance graph $G_{n}^{d}$ and a POI mobility connectivity $G_{n}^{m}$ , defined in Section 3.3) and a topic distribution **t**_*n*_ extracted from textual descriptions $E_{n}$ . Let ${\tilde{r}}_{n}$ be the embedding vector of the n-th entity. The objective is to optimize a function that measures representation loss and feature-topic alignment:

$$\tilde{R}={argmax}_{{\tilde{r}}_{n}}\overset{N}{\underset{n=1}{\sum }}f\left({\tilde{r}}_{n}|t_{n},G_{n}^{d},G_{n}^{m},K\right),$$ (1)

where $\tilde{R}={\left\{{\tilde{r}}_{n}\right\}}_{n=1}^{N}\in R^{N\times K}$ are the embeddings of all spatial entities, *K* is the number features of an embedding vector.

## 3 The Proposed Method-AutoFTP

In this section, we first introduce an overview of our AutoFTP framework, then present its technical details.

### 3.1 Framework Overview

**FIGURE 5:**
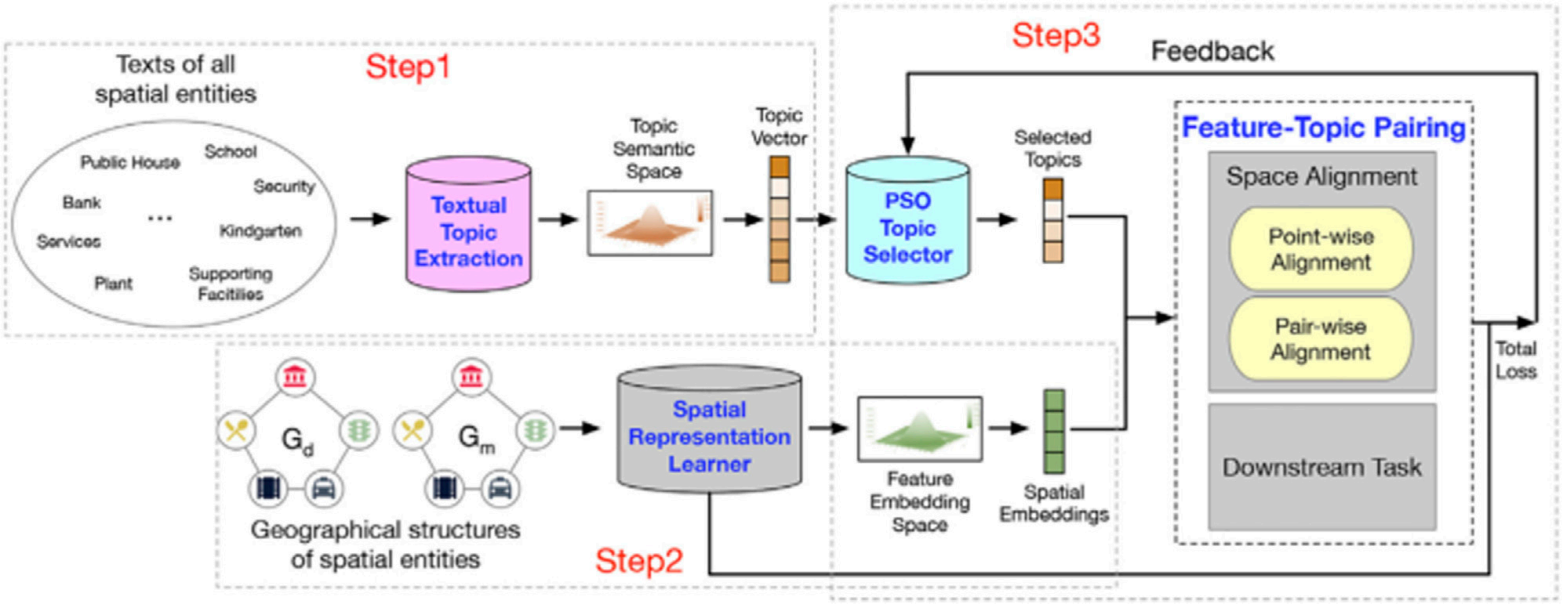
An overview of AutoFTP. In the framework, we first construct a topic semantic space based on the texts of spatial entities. Then, we initialize a embedding feature space based on the geographical structures of spatial entities. Later, we employ a PSO-based framework to conduct feature-topic pairing through jointly optimizing representation learning, point-wise alignment, pair-wise alignment, and downstream task over learning iterations.

Figure 5 shows our proposed framework. First, we construct a semantic topic space by extracting topic distribution from the corresponding texts of spatial entities. Then, we initialize a feature embedding space based on the geographical structures of spatial entities. Next, we utilize a PSO-based topic selector to select the optimal K topics for pairing with the spatial embeddings coming from the feature embedding space. During the pairing process, the losses of spatial representation learner, point-wise alignment, pair-wise alignment, and downstream tasks are regarded as feedback to update the topic selector for the next pairing iteration. With the development of the learning iteration, the feature embedding space aligns to the topic semantic space gradually. Finally, the learned spatial embeddings of AutoFTP are effective and semantically rich. Here, to validate the effectiveness of AutoFTP, we apply the framework to predict the real estate price (downstream tasks) of the residential communities (spatial entities) based on spatial embeddings of the communities. The more accurate the prediction is, the more effective the learned embedding is. In addition, the AutoFTP can be generalized to other spatial representation learning problems with graphs and texts.

### 3.2 Textual Topic Extraction

To derive the textual semantic topic space, we extract the topic distributions of spatial entities from texts generated by location based social networks. Traditional topic models, such as LDA (Blei et al., 2003), PSLA (Hofmann, 2013), are implemented based on bag-of-words. These methods ignore word orders in sentences. To improve the performances of topic modeling, we employ a pre-trained deep word embedding model (He, 2014) to generate topics.

**FIGURE 6:**
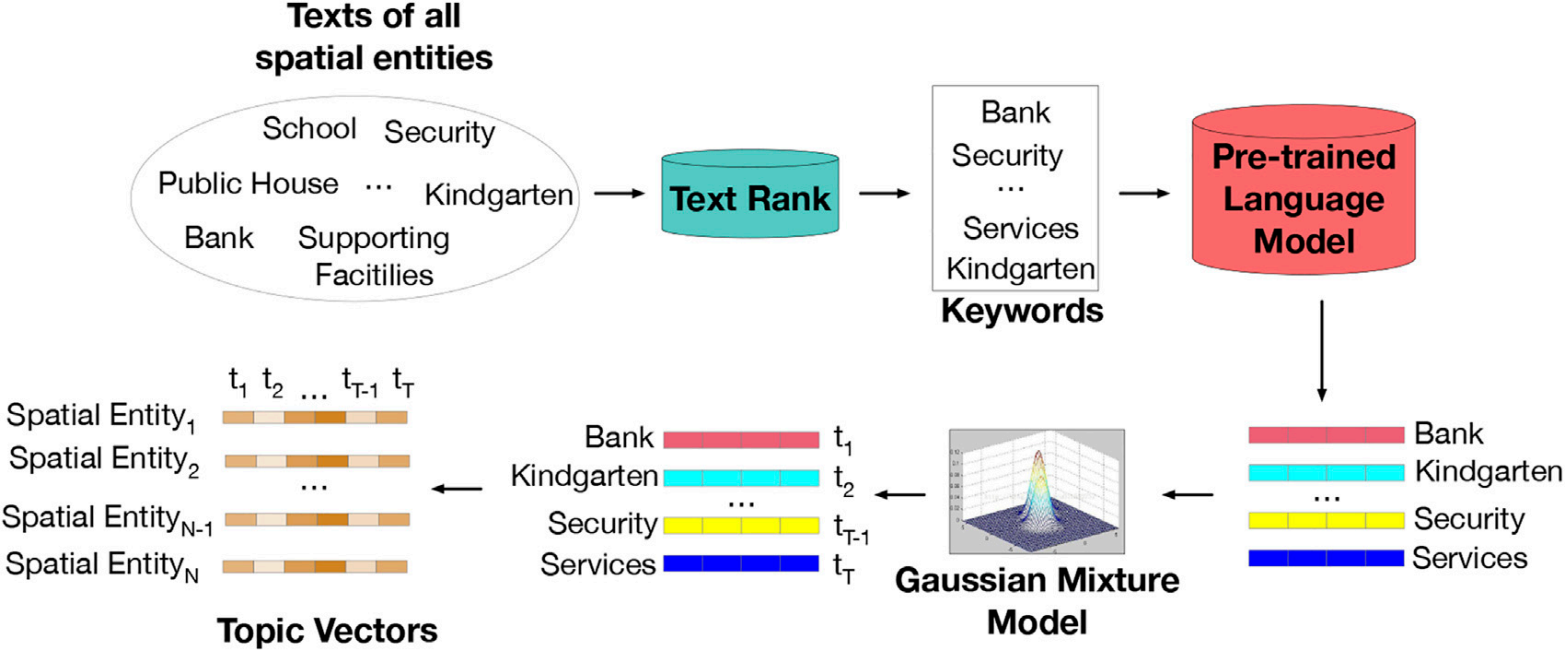
Obtaining the topic vectors of spatial entities.

As illustrated in Figure 6, we first collect the text descriptions of all entities. Besides, we extract keywords from texts using the TextRank algorithm (Mihalcea and Tarau, 2004) and leverage a pre-trained language model (He, 2014) to learn the corresponding word embedding of each keyword. Moreover, we exploit a Gaussian Mixture Model (GMM) to cluster the keyword embeddings into *T* topics. The clustering model provides a topic label for each keyword. To explain the labeling process, we take the *i*-th keyword’s embedding vector **x**_*i*_ as an example. First, we assume that the *T* topics obey a Gaussian Mixture Distribution (GMD). Then we randomly initialize the parameters of GMD. Next, we use the Expectation Maximization (EM) algorithm to find the optimal parameters of the GMD. Finally, we calculate the probability of **x**_*i*_ (a.k.a., membership), belonging to each topic based on the GMD, and select the topic with the highest probability as the label of **x**_*i*_. After that, we propose to construct the topic distribution vector of each spatial entity. In particular, for the *n*-th entity, the topic vector **t**_*n*_ is a *T* dimensional vector, where each dimension indicates a topic, and is filled by the number of associated keywords.

### 3.3 Graph Extraction of Spatial Entities

In order to learn the embedding feature vectors of spatial entities, we propose to construct the graph-structured topology of each spatial entity. This is because there is inherent spatial autocorrelation between each two spatial entities, according to the geographical first law. We describe a spatial entity in terms of its POIs, by building two graphs. 1) POI-POI distance graph: denoted by *G* ^*d*^, where POI categories are nodes and the average distances between POI categories are edge weights. 2) **POI-**POI mobility graph: denoted by *G* ^*m*^, where nodes are POI categories, and edge weights are human mobility connectivity. The number of POI categories in this paper is *M*, and the two graphs are extracted via the method in (Wang et al., 2018a). Specifically, we first use a parametric function to estimate POI visit probability based on a taxi GPS trace data: $P\left(\varsigma \right)=\frac{\beta_{1}}{\beta_{2}}\cdot \varsigma \cdot exp\left(1-\frac{\varsigma }{\beta_{2}}\right),$ where *ς* denotes the distance between a POI and a drop-off position in a taxi trace, *β*_1_ =  max_*ς*_ *P*(*ς*), and *β*_2_ = arg max_*ς*_ *P*(*ς*). We calculate the visited probability of all POIs according to the formula. We sum up the probability of POIs belonging to the same POI category to calculate the visited probability of the POI category. Finally, we calculate the connectivity strength between POI categories as: $C_{\vec{ij}}=\left\{\begin{array}{c} P_{i}\cdot P_{j},\;if\;\;i\neq j\;\\ 0,\;\;\;otherwise,\; \end{array}\right.$ where *P*_*i*_ and *P*_*j*_ represent the visited probability of POI category *i* and POI category *j* respectively; $C_{\vec{ij}}$ indicates the connectivity between POI category *i* and *j*.

### 3.4 Particle Swarm Optimization Based Feature-Topic Pairing

#### 3.4.1 Spatial Representation Learner

To learn the representations of spatial entities, we utilize the Graph Auto Encoder (GAE) (Kipf and Welling, 2016) to construct latent embedding space. Specifically, to learn the embedding feature vector of the *n*-th entity, the encoder has two GCN layers. The encoding calculation process can be formulated as follows:

$$\left\{\begin{array}{c} {\hat{A}}_{n}=A_{n}+I_{n},\;\\ {\tilde{A}}_{n}={\hat{D}}_{n}^{-\frac{1}{2}}{\hat{A}}_{n}{\hat{D}}_{n}^{-\frac{1}{2}},\;\\ z_{n}={\tilde{A}}_{n}\text{Relu}\left({\tilde{A}}_{n}X_{n}W_{n}^{\left(0\right)}\right)W_{n}^{\left(1\right)}\; \end{array}\right.$$ (2)

where $A_{n},I_{n},{\tilde{A}}_{n},{\hat{D}}_{n}$ own the same shape $R^{M\times M}$ . Moreover, **A**_*n*_ is the adjacency matrix, **I**_*n*_ is the identity matrix, ${\tilde{A}}_{n}$ is the symmetrically normalized adjacency matrix, ${\hat{D}}_{n}$ is the degree matrix. In addition, $X_{n}\in R^{M\times U}$ is the feature matrix of the graph, in which *U* is the feature dimension; $W_{n}^{\left(1\right)}\in R^{U\times H}$ is the weight matrix of the first GCN layer, in which *H* is the output dimension of the layer; $W_{n}^{\left(2\right)}\in R^{H\times K}$ is the weight matrix of the second GCN layer; $z_{n}\in R^{M\times K}$ is the output embedding of the encoder. The decoder recovers the adjacency matrix according to **z**_*n*_:

$${\hat{A}}^{*}=\sigma \left(z_{n}z_{n}^{\prime }\right).$$ (3)

The optimization objective is to minimize the reconstruction loss between the original graph, denoted by the adjacency matrix ${\hat{A}}_{n}$ , and the reconstructed graph, denoted by the adjacency matrix ${\hat{A}}_{n}^{*}$ :

$$L_{R}=\overset{N}{\underset{n=1}{\sum }}\|\left({\hat{A}}_{n}-{\hat{A}}_{n}^{*}\right){\|}^{2}$$ (4)

We apply the GAE to the POI-POI distance graph $G_{n}^{d}$ and the POI-POI mobility graph $G_{n}^{m}$ of the *n*-th spatial entity. After that, we obtain the node representations of $G_{n}^{d}$ and $G_{n}^{m}$ , denoted by $z_{n^{d}}\in R^{M\times K}$ and $z_{n^{m}}\in R^{M\times K}$ . Then, we aggregate $z_{n^{d}}$ and $z_{n^{m}}$ by averaging all node embeddings together to attain the graph embedding of $G_{n}^{d}$ and $G_{n}^{m}$ respectively. Finally, we integrate the graph embeddings of $G_{n}^{d}$ and $G_{n}^{m}$ into the unified spatial embedding of the entity by averaging calculation, denoted by $r_{n}\in R^{K}$ .

#### 3.4.2 Measuring the Alignment of Embedding and Semantic Spaces

To pair features with topics, we conduct space alignment from the point-wise and pair-wise perspectives. Referring to definitions Section 2.2 and Section 2.3, we aim to align the topic semantic space and feature embedding space from the coordinate system and information contents respectively. During the aligning process, we minimize the point-wise alignment loss $L_{P}$ and pair-wise alignment loss $L_{C}$ . To be convenient, we take the *n*-th entity as an example to explain the calculation process.

1) Point-wise Alignment Loss: $L_{P}$ *.* We first select *K* values from the topic vector **t**_*n*_ as the vector ${\overset{ˇ}{t}}_{n}\in R^{K}$ , which contains the most representative semantics in the semantic space. Then, we maximize the correlation between ${\overset{ˇ}{t}}_{n}$ and the spatial embedding **r**_*n*_, which is equal to minimize the negative correlation between the two vectors. The formula of the minimizing process as follows:

$$L_{P}=-\overset{N}{\underset{n=1}{\sum }}\frac{\text{cov}\left({\overset{ˇ}{t}}_{n},r_{n}\right)}{\delta \left({\overset{ˇ}{t}}_{n}\right)\delta \left(r_{n}\right)},$$ (5)

where cov(.) denotes the covariance calculation; *δ*(.) denotes the standard deviation.

2) Pair-wise Alignment Loss: $L_{C}$ *.* We first construct the topic-topic similarity matrix **S** and the feature-feature similarity matrix **S**′. Specifically, for $S\in R^{K\times K}$ , we calculate the similarity between any two topics. For $S^{\prime }\in R^{K\times K}$ , we calculate the similarity between two features of spatial embeddings. We keep the pair-wise consistency between **S** and **S**′ by minimizing the Frobenius norm, as follows:

$$L_{C}=\|S-S\prime {\|}_{F}.$$ (6)

#### 3.4.3 Supervised PSO For Automatic Topic Selection

As introduced above, we select *K* topics so the representation learner can learn a *K*-sized embedding vector in terms of *K* topics to achieve feature-topic alignment. However, how can the machine automatically identify the best *K* and select the most appropriate *K* topics?

A naive idea is that we can select *K* topics randomly at each iteration until we traverse all topic combinations and find the best topic subset based on the objective function. The searching process, however, is time-consuming and computationally expensive. Moreover, the topic selection problem belongs to the combinatorial optimization field, which is hard to solve by derivative-based optimization algorithms. Thus, a quickly and derivative-free optimization algorithm should be selected as our optimizer. Considering the high time complexity for traversing all possible subsets to find the optimal result, we propose to formulate the joint task of feature learning, topic selection, topic and feature matching into a PSO problem.

**FIGURE 7:**
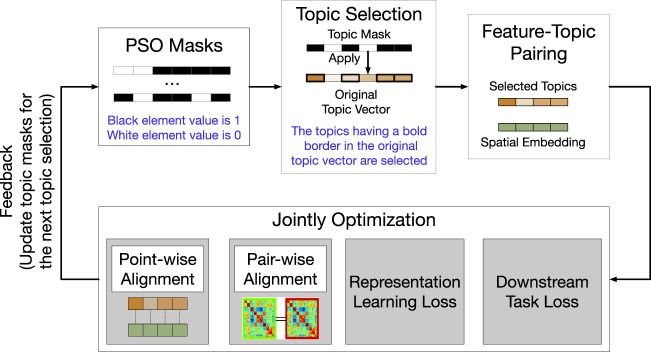
The optimization process of the PSO topic selector.

The PSO-based optimization framework is as illustrated in Figure 7. Specifically, we first randomly initialize a number of particles in PSO, where a particle is a binary topic mask (i.e., the mask value of 1 indicates “select” and the mask value of 0 indicates “deselect”). In other words, a set of particles select a subset of topics. A multi-objective deep learning model, whose objective function includes the losses of graph reconstruction, semantic alignment, and the regression estimator in the downstream task, is trained to learn spatial representations, using each selected topic subset. As an application, we use the embedding of spatial entities (residential communities) to predict their real estate prices, and the loss of the regression model $L_{Reg}$ is:

$$L_{Reg}=\frac{1}{N}\overset{N}{\underset{n=1}{\sum }}{\left(c_{n}-c_{n}^{*}\right)}^{2},$$ (7)

where *c*_*n*_ is the golden standard real estate price and $c_{n}^{*}$ is the predicted price. Next, we calculate the fitness of each particle according to the total loss of the deep model. The fitness can be calculated by:

$$Fitness=L_{C}+L_{P}+L_{R}+L_{Reg}.$$ (8)

Then, we utilize the fitness to inform all particles how far they are from the best solution. Next, each particle moves forward to the solution based on not only its current status but also all particles’ movement. After the fitness value of PSO converges, PSO identifies the best topic subset. Finally, the semantically-rich embeddings of spatial entities, given by: $\tilde{R}={\left\{{\tilde{r}}_{n}\right\}}_{n=1}^{N}$ .

## 4 Experimental Results

In this section, we present extensive experiments with real world data to answer the following research questions: Q1. How effective is our proposed AutoFTP in spatial representation learning? Q2. How effective is each loss function of AutoFTP? Are they all necessary for spatial representation learning? Q3. How well does AutoFTP work in space alignment? Do the learned spatial embeddings contain semantic meanings? Q4. How about the robustness of AutoFTP? Does it always outperform other baselines? Q5. How about the stability and sensitivity of AutoFTP?

### 4.1 Experimental Setup

#### 4.1.1 Data Description

Table 1 shows the statistics of five data sources used in the experiments. Firstly, the taxi traces data describes the GPS trajectory of taxis in Beijing in 3 months. The format of each trace record is < trip id, distance, travel time, average speed, pick-up time, drop-off time, pick-up location, drop-off location >. Secondly, the residential regions, texts, and real estate price data sources are crawled from www.fang.com. In experiments, the residential regions are treated as spatial entities. The texts reflect the urban utilities and characteristics of spatial entities from multiple perspectives such as traffic condition, economic development, demographic situation, and etc. The real estate prices indicate the average value of the real estate of each spatial entity in 6 months. Thirdly, the POIs are extracted from www.dianping.com, which is a POI (small businesses such as restaurants, banks, gas stations, shopping markets) review website in China. Each POI is described in a format of < POI id, POI category, latitude, longitude >.

**TABLE 1:** Statistics of the experimental data.

Data sources	Properties	Statistics
Taxi Traces	Number of taxis	13,597
	Time period	Apr.–Aug. 2012
Residential regions	Number of residential regions	2,990
	Time period of transactions	04/2011–09/2012
POIs	Number of POIs	328,668
	Number of POI categories	20
Texts	Number of textual descriptions	2,990
	Time Period	04/2011–09/2012
Real Estate Prices	Number of real estate prices	41,753
	Time Period	12/2011–06/2012

#### 4.1.2 Application: Real Estate Price Prediction

Our proposed method (AutoFTP) can learn a list of vectorized representations for all spatial entities. Therefore, as a downstream application, we can apply these representations to train a regression model to predict the average real estate price of these spatial entities. Specifically, we first apply AutoFTP to learn a series of representations of spatial entities based on their geographical structural information and related text descriptions. Then, we build up a deep neural network (DNN) model for predicting average real estate price of each spatial entity according to its corresponding representation. To be convenient, we take the *n*-th spatial entity as an example to explain the regression model. The formulation of DNN is $f\left({\tilde{r}}_{n},w\right)=w\cdot g\left({\tilde{r}}_{n}\right)+b$ , where ${\tilde{r}}_{n}$ is the representation of the *n*-th spatial entity, $g\left({\tilde{r}}_{n}\right)$ is the nonlinear transformation of ${\tilde{r}}_{n}$ , **w** is the weight term, and **b** is the bias term. We want to minimize the difference between predicted price $f\left({\tilde{r}}_{n},w\right)$ and real price *y*_*n*_. Thus, the objective of the DNN is $\text{min}\frac{1}{N}\sum_{n=1}^{N}{\left(y_{n}-f\left({\tilde{r}}_{n},w\right)\right)}^{2}$ , where *N* is the total number of spatial entities.

#### 4.1.3 Evaluation Metrics

We evaluated our method using a real estate price prediction task (Section 4.1.2). We took the feature representation vectors of residential communities as inputs, and predicted their real estate prices. We compared the golden-standard prices *y*_*n*_ with the predicted prices ${\hat{y}}_{n}$ in terms of four metrics: 1) $\text{RMSE}=\sqrt{\frac{1}{N}\sum_{n=1}^{N}{\left(y_{n}-{\hat{y}}_{n}\right)}^{2}}$ ; 2) $\text{MAE}=\frac{1}{N}\;\sum_{n=1}^{N}\left|\left(y_{n}-{\hat{y}}_{n}\right)\right|$ ; 3) $\text{MAPE}=\frac{100}{N}\sum_{n=1}^{N}\left|\frac{y_{n}-\hat{y_{n}}}{y_{n}}\right|$ ; 4) $\text{MSLE}=\frac{1}{N}\sum_{n=1}^{N}{\left(log\left(1+y_{n}\right)-log\left({\hat{y}}_{n}+1\right)\right)}^{2}$ . The regression loss and optimization algorithm are controlled to be the same. The lower the four metrics are, the more effective the spatial embedding features are.

#### 4.1.4 Baseline Algorithms

We compared our proposed method with seven widely-used and robust representation learning (embedding) methods as follows: 1) AttentionWalk (Abu-El-Haija et al., 2018) utilizes a novel attention model to automatically learn the hyper-parameters of random-walk based network embedding methods, which improves the flexibility and performance of the model. We set the learning rate as 0.01, the regularization parameters as 0.5. 2) ProNE (Zhang J. et al., 2019) formulates the network embedding as sparse matrix factorization to improve the calculation speed, and conducts the propagation process in the spectrally modulated space to enhance the representation. We adopt the default parameter setting in (Zhang J. et al., 2019). 3) GatNE (Cen et al., 2019) is a random-walk based network embedding method, which considers the information of different attributes of nodes to enhance the graph representation. We set the number of walks as 20, walk length as 10, window size as 5, patience as 5. 4) GAE (Kipf and Welling, 2016) utilizes GCN to learn the node representations in the encode-decoder paradigm by minimizing the reconstruction loss. We set the number of GCN layers as 2 and the learning rate as 0.0001. 5) DeepWalk (Perozzi et al., 2014) is an extension of the word2vec model (Mikolov et al., 2013), which brings the idea of truncated random walks to a network embedding scenario. We set the number of walks as 80, walk length as 10, and window size as 5. 6) Node2Vec (Grover and Leskovec, 2016) is an enhanced version of DeepWalk, which considers the homogeneity and structural equivalence of networks during embedding process. We set the number of walks as 80, walk length as 10, window size as 5, return parameter *p* as 0.25 and in-out parameter *q* as 4. 7) Struc2Vec (Ribeiro et al., 2017) learns the node representation by considering the structural identity of nodes in the network. We set the number of walks as 80 and walk length as 10.

Besides, there are four losses in AutoFTP: reconstruction loss $L_{R}$ , point-wise alignment loss $L_{P}$ , pair-wise alignment loss $L_{C}$ , and regression loss $L_{Reg}$ . The four losses provide the optimization direction of AutoFTP. To study the benefits of each part, we develop four internal variants of AutoFTP: 1) **AutoFTP** ^*R*^, which only keeps $L_{R}$ of AutoFTP; 2) **AutoFTP** ^(*R*+*P*)^, which keeps $L_{R}$ and $L_{P}$ of AutoFTP; 3) **AutoFTP** ^(*R*+*C*)^, which keeps $L_{R}$ and $L_{C}$ of AutoFTP; 4) **AutoFTP** ^(*R*+*P*+*C*)^, which keeps $L_{R}$ , $L_{P}$ , and $L_{C}$ of AutoFTP. The dimension of embeddings in all models is 20.

#### 4.1.5 Hyperparameters, Source Code, and Reproducibility

We detailed the hyperarameters and the steps of our algorithm in the Appendix. We released our code^4^ to help to reproduce experimental results.

#### 4.1.6 Environmental Settings

The experimental studies were conducted in the Ubuntu 18.04.3 LTS operating system, plus Intel(R) Core(TM) i9-9920X CPU@ 3.50GHz, 1 way SLI Titan RTX and 128GB of RAM, with the framework of Python 3.7.4, Tensorflow 2.0.0, and Pyswarm 1.3.0.

### 4.2 Overall Performance (Q1)

Table 2 shows the comparison of all the 11 models. As can be seen, AutoFTP, in overall, outperforms the baseline algorithms in terms of RMSE, MAE, MAPE and MSLE. A possible reason for this observation is that compared with other baseline algorithms, AutoFTP not just captures geographical structural information but also preserves rich semantics of spatial entity. Besides, the regression estimator (the downstream task) of AutoFTP provides a clear learning direction (accuracy) for spatial representation learning. Thus, in the downstream predictive task, the spatial embedding features learned by AutoFTP beats all baselines.

**TABLE 2:** Overall Performance with respect to RMSE, MAE, MAPE and MSLE. (The smaller value is, the better performance is).

	RMSE	Outperform	MAE	Outperform	MAPE	Outperform	MSLE	Outperform
AutoFTP	18.646	—	16.192	—	58.851	—	0.2267	—
AttentionWalk	21.418	+14.9*%*	19.712	+21.7*%*	68.590	+16.6*%*	0.2907	+28.2*%*
ProNE	21.830	+17.1*%*	19.929	+23.1*%*	69.188	+17.6*%*	0.2949	+30.1*%*
GatNE	21.229	+13.9*%*	19.288	+19.1*%*	67.043	+13.9*%*	0.2854	+25.9*%*
GAE	21.338	+14.4*%*	19.676	+21.5*%*	68.579	+16.5*%*	0.2894	+27.6*%*
DeepWalk	23.561	+26.4*%*	21.987	+35.8*%*	76.038	+29.2*%*	0.3321	+46.5*%*
Node2Vec	22.688	+21.7*%*	21.084	+30.2*%*	73.135	+24.3*%*	0.3152	+39.0*%*
Struc2Vec	21.589	+15.8*%*	19.937	+23.1*%*	69.423	+17.9*%*	0.2942	+29.7*%*
AutoFTP^*R*^	21.965	+17.8*%*	20.283	+25.3*%*	70.991	+20.6*%*	0.2928	+29.1*%*
AutoFTP^(*R*+*P*)^	20.509	+9.99*%*	18.921	+16.8*%*	66.477	+12.9*%*	0.2681	+18.3*%*
AutoFTP^(*R*+*C*)^	21.014	+12.7*%*	19.413	+19.8*%*	67.920	+15.4*%*	0.2773	+22.3*%*
AutoFTP^(*R*+*P*+*C*)^	20.211	+8.39*%*	18.676	+15.3*%*	65.685	+11.6*%*	0.2636	+16.3*%*

In addition, another interesting observation is that among all baseline models, GatNE outperforms others in terms of all evaluation metrics. Such observation shows that GatNE considers different attributed information of nodes in spatial graphs of spatial entities. Thus, the spatial embedding features learned by GatNE are more effective compared with other baseline models. Moreover, after further observing Table 2, we can find that the predictive performances of GAE are better than most random-walk based approaches, except GatNE. Such observation indicates that the graph convolution-based methods (GAE, AutoFTP) are more suitable than the random-walk based approaches (other baselines) in modeling geographical structure information. In summary, the overall performance experiment shows the superiority and effectiveness of AutoFTP compared with other baseline models.

### 4.3 Study of AutoFTP Variants (Q2)

To validate the necessity of each loss of AutoFTP, we internally compared the performances of AutoFTP with the performances of the variants of AutoFTP. Table 2 shows the ranking orders of the predictive accuracies of the compared methods are: AutoFTP > AutoFTP^(*R*+*P*+*C*)^ > AutoFTP^(*R*+*P*)^ > AutoFTP^(*R*+*C*)^ > AutoFTP^*R*^. A potential interpretation for the observation is that with the increase of optimization objective (loss), AutoFTP captures more characteristics of spatial entities from representation learning, point-wise alignment, pair-wise alignment, and regression task. In addition, compared with AutoFTP^(*R*+*P*)^ and AutoFTP^(*R*+*C*)^, we find that the predictive performance of AutoFTP^(*R*+*P*)^ is better than AutoFTP^(*R*+*C*)^. A plausible reason for the observation is that the features of spatial entities captured by point-wise alignment are more indicative for spatial entities compared with them learned by pair-wise alignment. Moreover, another interesting observation is that AutoFTP outperforms other variants by a large margin. Such observation indicates that the regression loss $L_{Reg}$ provides a clear optimization direction for AutoFTP, which preserves the features related to the downstream task into spatial embeddings. To sum up, the ablation study experiment demonstrates the four loss functions of AutoFTP are necessary for capturing the representative features in spatial entities during spatial representation learning process.

### 4.4 Study of the Interpretability of Spatial Embeddings (Q3)

The space alignment in AutoFTP is implemented from two perspectives: point-wise alignment and pair-wise alignment. The two kinds of alignment make the learned spatial embeddings contain more semantic meaning and interpretability.

#### 4.4.1 Study of the Point-wise Alignment

**FIGURE 8:**
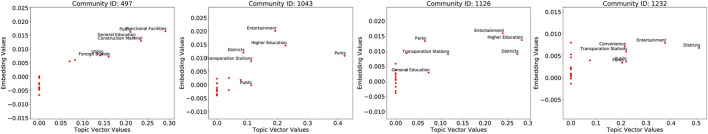
Illustration of Point-wise Alignment with sample communities.

To analyze the point-wise alignment, we picked communities (spatial entities) 497, 1,043, 1,126, and 1,232 as examples to plot their corresponding embedding vectors against their corresponding topic vectors. Meanwhile, we extracted the topic names of the most significant 6 topics. Figure 8 shows AutoFTP keeps the point-wise consistency between the semantic feature space and the embedding space. Moreover, the learned spatial embeddings contain abundant semantic meanings. We can infer the urban functions for each community based on Figure 8. For instance, the community #497 exhibits high weights on some specific topics, such as, functional facilities, general education, and construction materials. Such observation indicates that this community is probably a large residential area with well-decorated apartments and general education institutions. The community #1043 and #1126 all have high weights in entertainment, higher education, parks, etc. We can speculate that they are both residential regions nearby universities. This is because the facilities belonging to these topics indicates the two communities are very likely to be in a college town. For the community #1232, it exhibits high weights in district, entertainment and convenience related categories. We can infer that the community is a commercial district with many transportation facilities.

#### 4.4.2 Study of the Pair-wise Alignment

**FIGURE 9:**
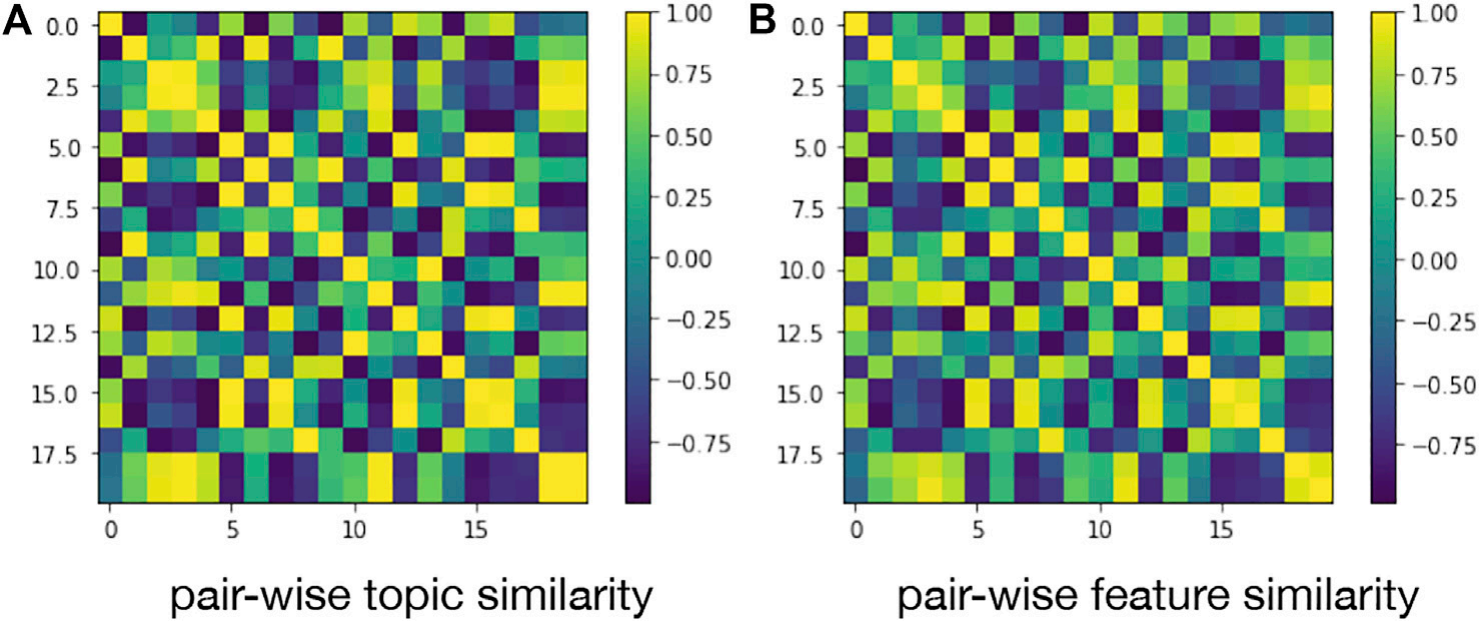
Illustration of pair-wise alignment.

To observe the pair-wise alignment, we visualized the pair-wise topic similarity matrix and pair-wise feature matrix by heat map respectively. As illustrated in Figure 9, we can find that the two matrices are similar with only minor differences. The observation indicates that the embedding feature space is well-matched with the semantic feature space.

#### 4.4.3 Study of the Interpretability

**FIGURE 10:**
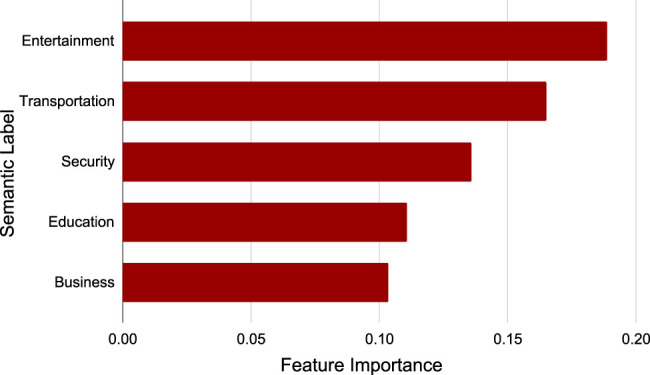
Feature importance of spatial embeddings.

The results of section 4.4.1 and section 4.4.2 shows that the feature embedding space and the topic semantic embedding space are aligned well. To study the interpretability of spatial embeddings further, we built up a tree model for real estate price prediction and then analyze the feature importance based on the semantic labels of the spatial embeddings. Specially, we exploited a random forest model to predict the real estate price of spatial entities based on the corresponding embeddings. Then, we collected the feature importance of the model as illustrated in Figure 10. We can find that the semantic labels of top 5 dimensions in the embeddings that affects the real estate price prediction are “Entertainment”, “Transportation”, “Security”, “Education”, and “Business”. The three most representative keywords in each semantic label, as shown in Table 3. In common sense, the 5 semantic labels are the most important factors that people consider for buying an estate (Boiko et al., 2020). In other words, they affect the real estate price heavily. Thus, the feature importance analysis experimental results are reasonable. In summary, this experiment validates that AutoFTP can select the most significant topic semantics for feature-topic automatically. In addition, the semantic labels of the spatial embeddings can be regarded as an auxiliary information to improve the interpretability of the embeddings.

**TABLE 3:** Semantic labels and top 3 keywords.

Semantic label	Keywords
Entertainment	Work Out, Tennis Court, Golf Court
Transportation Facilities	Highways, High Speed Rail, Bus Stations
Security	Surveillance, Firefighting, Emergency
Education	Primary School, High School, University
Business	Commercial Street, Canal, Satellite City

### 4.5 Robustness Check (Q4)

**FIGURE 11:**
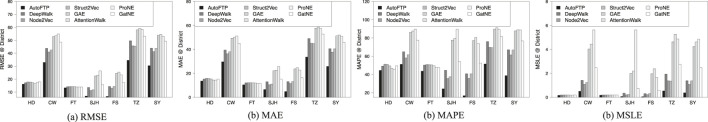
Robustness check according to geographical district.

To evaluate the robustness of AutoFTP, we divided the embeddings into 5 groups (HaiDian, ChongWen, FengTai, ShiJingShan, FangShan) according to the geographical district of spatial entities. Figure 11 shows that AutoFTP consistently outperforms the baselines, and performs more stably than the baselines across the five districts. Such observation indicates that AutoFTP captures the unique local features of different spatial groups. There are two possible reasons for the observation: 1) the semantic alignment of AutoFTP injects the distinct semantic characteristics of spatial entities into the learned embeddings; and 2) the customized regression estimator provides a clear optimization objective for AutoFTP. Overall, the robustness check experiment demonstrates that AutoFTP outperforms other baseline models in not only the global zone but also each local spatial sub-areas.

### 4.6 Study of the Stability and Sensitivity (Q5)

**FIGURE 12:**
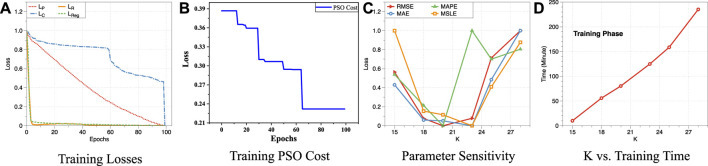
Study the stability and sensitivity of AutoFTP. **(A)** Training Losses. **(B)** Training PSO Cost. **(C)** Parameter Sensitivity. **(D)** K vs. Training Time.

In this section, we fully evaluated the stability and parameter sensitivity of AutoFTP. We first examined the stability of AutoFTP by analyzing the training losses of AutoFTP and convergence of PSO optimization part. To observe the changing trend of each loss objectively, we scaled the value of losses into [0 ∼ 1] and visualized them in Figure 12A. We can find that all losses (reconstruction loss $L_{R}$ , regression loss $L_{Reg}$ , point-wise loss $L_{P}$ , pair-wise loss $L_{C}$ ) reach convergence over training iterations. Especially, $L_{R}$ and $L_{Reg}$ reach equilibrium quickly only after 10 epochs. This observation validates the training stability of AutoFTP. We also analyzed the convergence of PSO. As shown in Figure 12B, the PSO optimization part reaches convergence after 65 epochs, which further indicates the stable performance of AutoFTP. For the parameter sensitivity evaluation, we investigated the influence of the parameter *K* (the dimension of final embeddings and the number of significant topics) for the model performance and the training time. The same to Figure 12A, we scaled the value of all metrics into [0 ∼ 1] and visualized them in Figure 12C. We can find that the value of *K* affects the model performance heavily. The observation is reasonable because *K* determines the information content of the final learned embeddings. The plots in Figure 12D show that the larger *K* is, the longer the training time is. A potential reason for the observation is that the larger *K* means that we need to try more topic subsets for feature-topic pairing.

## 5 Related Work

Graph Representation Learning with Latent Semantics. Graph representation learning refers to techniques that preserve the structural information of a graph into a low-dimensional vector (Wang et al., 2016; Abu-El-Haija et al., 2018; Zhang J. et al., 2019; Cen et al., 2019; Wang et al., 2020b). However, owing to traditional graph representation learning models are implemented by deep neural networks, the learned embeddings lack interpretability. Recently, to overcome this limitation, researchers leveraged the texts related to graphs to learn semantically rich representations. For instance, Mai et al. implemented an entity retrieval academic search engines that incorporate the text embedding and knowledge graph embedding for accelerating retrieving speed (Mai et al., 2018). Xiao et al. improved the semantic meaning of knowledge graph’s embedding by integrating both graph triplets and textual descriptions of spatial entities (Xiao et al., 2017). Different from these studies, in this paper, based on spatial entities data composing by spatial graphs and related texts, we propose a new representation learning framework that unifies feature embedding learning and feature-topic pairing together in a closed-loop manner by a PSO based optimization method.

Topic Models in Spatio-temporal Domain. Topic models aim to automatically cluster words and expressions patterns for characterizing documents (Xun et al., 2017; Lee and Kang, 2018; Hu et al., 2019). Recently, to understand the hidden semantics of spatial entities, many researchers applied topic models in the spatio-temporal data mining domain (Zheng et al., 2017; Huang et al., 2019; Huang et al., 2020). For instance, Zhao et al. discovered representative and interpretable human activity patterns from transit data automatically by a spatio-temporal topic model (Zhao et al., 2020). Yao et al. tracked spatio-temporal and semantic dynamics of urban geo-topics based on an improved dynamic topic model that embeds spatial factors of pairwise distances between tweets (Yao and Wang, 2020). These successful applications validate the effectiveness of topic models for extracting semantics in spatio-temporal domains. However, traditional topic models only focus on word frequency in texts but neglect the semantics of words. Recently, the success of many pre-trained language models (Vaswani et al., 2017; Kenton and Toutanova, 2019; Yang et al., 2019) brings hope for producing more reasonable topic distribution. Thus, in this paper, we employ a pre-trained language model to get the embeddings of keywords and utilize Gaussian Mixture Model to extract topic distribution based on the embeddings.

Explainable Artificial Intelligence (XAI) With artificial intelligence methods are applied in multiple scenarios successfully, how to improve the model explainability becomes a big challenge. In the traditional machine learning domain, researchers employ some simple models that own the explainability naturally such as linear models, decision trees, rule-based models, and etc to explain the modeling process (Burkart and Huber, 2021; Lakkaraju et al., 2016; Lakkaraju et al., 2017). For instance (Lundberg et al., 2020), improved the global interpretability of tree models by combining many local feature explanations of each prediction and obtained good performance on three medical machine learning problems by applying these models (Wang and Rudin, 2015). provided a Bayesian framework for learning falling rule lists that do not rely on traditional greedy decision tree learning approaches to improve the explainability of classification models. Although these approaches can improve the model interpretability, the model performance often is sacrificed. Recently, the excellent predictive performance of deep learning models leads the techniques have been applied in many scenarios such as fraud detection, credit evaluation, healthcare, etc. But explainability is the key limitation of the deep learning models. To improve the model explainability, XAI on deep learning attracts much attention from researchers (Gunning, 2017; Selvaraju et al., 2017; Samek and Müller, 2019; Agarwal et al., 2020). For instance (Selvaraju et al., 2017), proposed a gradient-weighted class activation mapping method to highlight the import regions in the image for predicting the concept. (Agarwal et al., 2020). proposed neural additive models that learns a linear combination of neural networks for depicting the complex relationships between input features and the output. However, these models focus on studying the relationship between the embeddings and outputs, but cannot provide explicit semantic meanings. Different from these studies, we try to give explicit semantic labels for the learned embeddings through the alignment between the feature embedding space and topic semantic space.

Comparison with Prior Literature As an emerging feature extraction technique, deep SRL has demonstrated the power in automated geographic and spatial feature extraction. However, SRL inherits drawbacks of traditional DNNs, such as: the embedding feature space lacks semantic interpretation. Texts can provide more interpretation, but spatial text mining has developed separately. Now, there is cross and increasing interests in both fields to benefit from the advances of the other. Our study targets at an unexplored area at the intersection between representation learning in geospatial data and topic label mining in texts. We develop and formulate a new problem: feature-topic pairing, to address the alignment challenges of the feature embedding space and the semantic topic space. The self-optimizing solution unifies representation learning, topic label selection, feature-topic matching in a PSO framework. This framework can be generalized to other integrated tasks, such as, representation learning integrated with not just topic based selection, but also causal selection, or other constrained selection over features, in various application senarios. This is how this study differentiates from and advances prior literature.

## 6 Conclusion

We presented a novel spatial representation learning (SRL) framework, namely AutoFTP. The spatial embeddings produced by traditional SRL models lack semantic meaning. To overcome this limitation, we formulated the feature-topic paring problem. We proposed a novel deep learning framework to unify representation learning, topic label selection, and feature-topic pairing. Specifically, we designed a segmentation-embedding-clustering method to generate candidate feature topic labels from texts. We developed an integrated measurement to measure the pointwise and pairwise alignment between topic label and embedding feature space. We devised a PSO based optimization algorithm to effectively solve the joint task of feature learning and feature-topic pairing. Our method integrated spatial graphs and associated texts to learn effective embedding features with visible labels. Extensive experiments demonstrated the effectiveness of AutoFTP by comparing it with other baseline models. The topic labels of the learned features were shown by many case studies and the feature importance analysis of a downstream task. For future work, we plan to extend our approach from geospatial networks to other applications that consist of graphs and texts, such as social media and software code safety.

## Data Availability

Publicly available datasets were analyzed in this study. This data can be found here: https://www.dropbox.com/sh/woqh4qvuzq1788r/AAB5Vz1DSeJiLKxq-POHLMAVa?dl=0.

## Footnotes

1
https://www.brookings.edu/blog/techtank/2016/08/18/autopilot-fatality-reveals-risks-of-technology-testing/

2
https://www.wsj.com/articles/BL-DGB-42522

3
https://www.propublica.org/article/machine-bias-risk-assessments-in-criminal-sentencing

4
https://www.dropbox.com/sh/cst7yegcp1yvwax/AAB_3xBtgGDod10ULgncI-4La?dl=0

## Appendix

### Reproducing the Algorithm

To claim AutoFTP clearly, we provide the pseudo-code of the learning process of AutoFTP. As illustrated in **Algorithm 1**, the framework includes three steps: (i) initializing the parameters of PSO, (ii) optimizing multiple objectives of AutoFTP, (iii) and outputting the final spatial embeddings. The framework takes topic vectors, POI-POI distance graphs, and POI-POI mobility graphs of spatial entities as input, and final semantically-rich embeddings as output.

For initializing the parameters of PSO (Line 1–5 in Algorithm 1), we first generate *M* particles as a particle swarm. Then, we initialize the position (topic mask) and velocity of each particle. Specifically, we sample *K* values from the uniform distribution *U*(0, 1) as the position vector, and sample *K* values from the uniform distribution *U*(−1, 1) as the velocity vector. Next, we update each particle’s best known position (pBest) and the swarm’s best known position (gBest) based on each particle’s position.

**Algorithm 1:** Automatic Feature-Topic Pairing (AutoFTP).

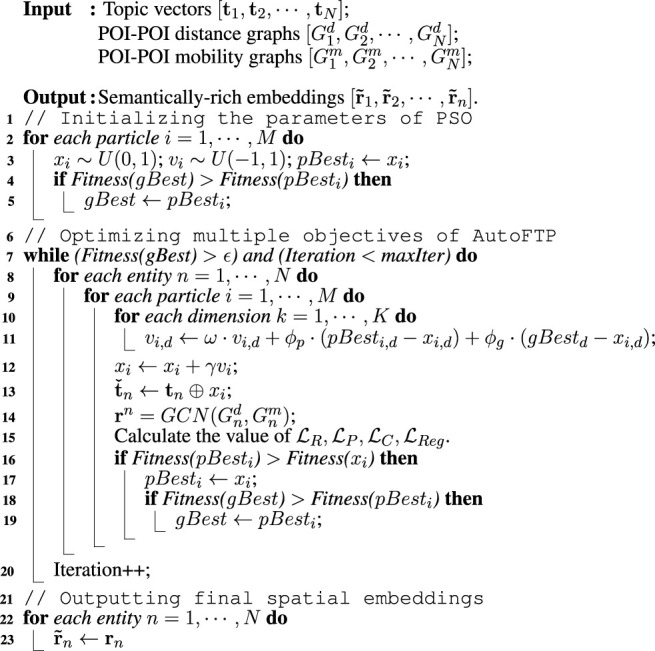

For optimizing multiple objectives of AutoFTP (Line 6–20 in Algorithm 1), we first check that if the optimization process achieves the termination conditions. If best topic mask is not found or the training iteration does not surpass the max iteration limitation, we optimize the objectives continually. Otherwise, we output the final spatial representations. During the optimization process, for one iteration, we utilize one particle to do feature-topic pairing. Specifically, we first update the velocity of the particle based on the old velocity, the gap between the current position and pBest, and the gap between the current position and gBest. Then, we generate a new position vector (topic mask) based on the velocity vector (Line 10–11 in Algorithm 1). In the two lines, *ω*, *ϕ*_*p*_, *ϕ*_*g*_ are weights, and *γ* is the learning rate of the corresponding items. Then, we filter *K* topics by the topic mask and generate the basic embedding of a spatial entity. In addition, we align the semantics of the *K* topics and the features of the basic embedding, and accomplish a downstream task simultaneously (Line 12–15 in Algorithm 1). Moreover, we evaluate the performance of the particle, and update the value of pBest and gBest for next optimization iteration (Line 16–20, in Algorithm 1).

For outputting final spatial embeddings (Line 21–23 in Algorithm 1), we copy the learned spatial representation as the final semantically-rich representations of spatial entities.
